# A Systematic Review: Efficacy of Different Intraocular Pressure-Lowering Agents in Black Individuals

**DOI:** 10.7759/cureus.86945

**Published:** 2025-06-29

**Authors:** Risantini Murugan, Veylamuthen Murugan, Pankaj K Agarwal

**Affiliations:** 1 Oncology, Queen Elizabeth Hospital Birmingham, Birmingham, GBR; 2 Internal Medicine, Russells Hall Hospital, Dudley, GBR; 3 Ophthalmology, Princess Alexandra Eye Pavilion, Edinburgh, GBR

**Keywords:** african american/black, ocular hypertension, open angle-glaucoma, prostaglandin analogue, raised intra-ocular pressure

## Abstract

Glaucoma is one of the leading causes of blindness worldwide, with the Black population experiencing an earlier onset and severe form of the disease compared to the White population. Despite the increased prevalence in the Black community, most research pertaining to glaucoma is focused on White patients. The current management guidelines recommend prostaglandin analogues and non-selective beta-adrenergic antagonists as the mainstay treatment, however their comparative efficacy in the Black community has not been thoroughly investigated.

This systematic review aims to investigate the efficacy of the various intraocular pressure (IOP) lowering agents used to treat primary open angle glaucoma (POAG) and ocular hypertension (OHT) in Black patients.

A systematic review of three randomised controlled trials was conducted to assess the efficacy of IOP-lowering agents specifically in the Black population. The protocol was set prior to the literature search and registered in PROSPERO (CRD420025652374). Databases searched included PubMed, Cochrane Library, Europe PMC and Semantic Scholar. Two independent researchers reviewed the articles and data was extracted from the three eligible articles. Risk of bias was assessed using the Risk of Bias (RoB) 2 tool.

Our study found prostaglandin analogues to be highly efficient in the Black community and non-selective beta-adrenergic antagonists to have a lower efficacy in this population. The results align with the current National Institute for Health and Care Excellence (NICE) guidelines recommending prostaglandin analogue as the first-line treatment for POAG and OHT. However, the NICE guidelines recommend non-selective beta-adrenergic antagonists as the second-line treatment, which has a lower efficacy in Black patients. In addition, our study found prostamides to be only slightly less efficient compared to prostaglandin analogues in the Black population.

In conclusion, prostaglandin analogues are a suitable first-line IOP-lowering agent in Black patients. Further research is required to investigate a suitable second-line treatment option in this community if patients have any adverse effects or do not tolerate the first-line option or need additional reduction in IOP.

## Introduction and background

Glaucoma is a progressive ocular condition resulting in optic neuropathy, with increased intraocular pressure (IOP) being a modifiable risk factor [1]. Studies have found various other risk factors that make a person more likely to have glaucoma, which are age, family history, corneal thickness, ethnicity, trauma to the eye, certain medications and medical conditions [2]. In a hospital setting, the IOP of the eye, visual field, optic nerve and retina are assessed to measure disease activity. Following diagnosis, clinicians would decide on the most appropriate management for the patient, considering topical treatments, lasers and surgical management [3].

A study done in 2013 investigating several African ethnic groups (Bantu subgroups, Hausa-Fulani, Ibo, Akwapim, Ewe, Akim, Ga-Adangbe, Ikpeye) and African-derived populations (African-Americans and African-Carribeans), found increased severity in glaucoma progression in Black patients and earlier onset of blindness in this population compared to White patients [4]. This study did not find a consistent association between gender and prevalence of open angle glaucoma [4]. The cause of the increased frequency and premature occurrence of this condition in this population is still unknown, however, some studies have found that certain genetic mutations such as the myocilin gene and the variations of the gene CDKN2B to be potential factors [5,6]. In addition, a lower corneal hysteresis has been associated with an increased risk of glaucoma progression with faster rates of visual field loss. In relation to that, African-Americans have been found to have lower corneal hysteresis compared to other ethnic groups [7,8].

The medical treatments available for this condition are different classes of IOP-lowering agents such as topical prostaglandin analogues, beta-adrenergic antagonists, carbonic anhydrase inhibitors, prostamides, miotics and sympathomimetics. The current National Institute for Health and Care Excellence (NICE) guidelines recommend prostaglandin analogues as first-line ongoing treatment for primary open angle glaucoma (POAG) and ocular hypertension (OHT) [9]. Our study aims to evaluate the efficacy of the various IOP-lowering agents in the Black population. A systematic review was conducted, involving all relevant published randomised controlled trials (RCTs) in the treatment of POAG and OHT in this population.

## Review

Methods

Objectives, Registration and Protocol

The updated Preferred Reporting Items for Systematic reviews and Meta-Analyses (PRISMA) 2020 recommendations were used to guide systematic literature search, abstract and full-text screening and selection of inclusion and exclusion criteria [10]. PICOS (participants/ population, interventions, comparisons, outcomes, and study design) question was formulated to understand the efficacy of different IOP-lowering agents in Black individuals.

Study Criteria

The protocol was set prior to the literature search and registered in PROSPERO (CRD420025652374) [11]. The inclusion and exclusion criteria are as shown in Table 1.

**Table 1:** Inclusion and exclusion criteria of the systematic review.

Inclusion Criteria	Exclusion Criteria
English-language	Studies on surgery-based interventions
Randomised-controlled trials	Studies lacking racial subgroup analysis
Black population	Studies of other languages
Medical history of primary open angle glaucoma or ocular hypertension	Case studies, case reports, case series
Studies comparing intraocular pressure lowering agents	

Information Sources

The literature search was performed on the most relevant scientific bases for the research question, i.e. PubMed, Cochrane Library, Europe PMC and Semantic Scholar. The computerised searches covered the period between January 1, 2004, and December 31, 2024.

Search Strategy

The following medical and subject heading (MeSH) terms keywords and modifications were combined in search strings using the Boolean operator “AND”: “primary open-angle glaucoma”, “ocular hypertension”, “glaucoma”, “prostaglandin analogues”, “latanoprost”, “bimatoprost”, “travoprost”, “timolol”, “dorzolamide”, “brinzolamide”, “intraocular pressure”, “tonometry”, “Black or African American” [Mesh], “African American”, “Black patients”, “African descent”, “Black race”. Database filters were utilised to filter only randomised-controlled trials published between 2004 and 2024.

The systematic search query as conducted in PubMed is (("primary open-angle glaucoma"[Title/Abstract] OR "ocular hypertension"[Title/Abstract] OR glaucoma[Title/Abstract] OR "glaucoma"[MeSH Terms]) AND ("prostaglandin analogues"[Title/Abstract] OR latanoprost[Title/Abstract] OR bimatoprost[Title/Abstract] OR travoprost[Title/Abstract] OR timolol[Title/Abstract] OR dorzolamide[Title/Abstract] OR brinzolamide[Title/Abstract]) AND ("intraocular pressure"[Title/Abstract] OR IOP[Title/Abstract]) AND ("Black"[Title/Abstract] OR "African American"[Title/Abstract] OR "African descent"[Title/Abstract] OR "Black patients"[Title/Abstract] OR "Black race"[Title/Abstract] OR "Black or African American"[MeSH Terms] OR "Afro-Caribbean"[Title/Abstract] OR "Sub-Saharan African"[Title/Abstract] OR "Sub-Saharan descent"[Title/Abstract])) AND (randomized controlled trial[Publication Type] OR randomized[Title/Abstract] OR RCT[Title/Abstract]) AND ("2004/01/01"[Date - Publication] : "2024/12/31"[Date - Publication]).

Selection of Studies and Extraction of Data

Two reviewers independently determined the study eligibility based on the inclusion and exclusion criteria. Duplicate records were removed by the AI-powered systematic review managing platform Rayyan (https://rayyan.ai). The titles and abstracts were screened first. The full text of included studies was then retrieved from the NHS Library Knowledge Hub and assessed for inclusion. Data were extracted from the text, tables and diagrams of included studies. Data extracted included design of the study, diagnosis, populations studied, sample size of the Black participants, evidence of central cornea thickness measurement, presence of a washout period, consideration of diurnal variation in IOP, IOP-lowering agents studied and the primary outcomes.

Data Synthesis, Assessment of the Risk of Bias and Critical Appraisal

The synthesis of the results followed the Cochrane and PRISMA guidelines. The assessment of the risk of bias (RoB) and the quality of the retrieved studies was conducted using the Cochrane RoB 2 for randomised-control trials considering the following domains: (1) Randomisation bias - ensuring random participant allocation; (2) Intervention bias - ensuring participants were blinded appropriately to the interventions; (3) Missing outcome data - evaluates impact of missing data; (4) Measurement bias - ensuring investigators were blinded and reliable measurement methods; (5) Reporting bias - ensures all outcomes were reported and there were no selective reporting [12]. The graphical representation of the RoB 2 assessment was produced using the robvis visualisation tool [13]. A meta-analysis was not performed due to the limited number of studies included (n=3) and the substantial heterogeneity in sample sizes and study methods in these studies.

Results

Study Selection and PRISMA Flow Diagram

**Figure 1:**
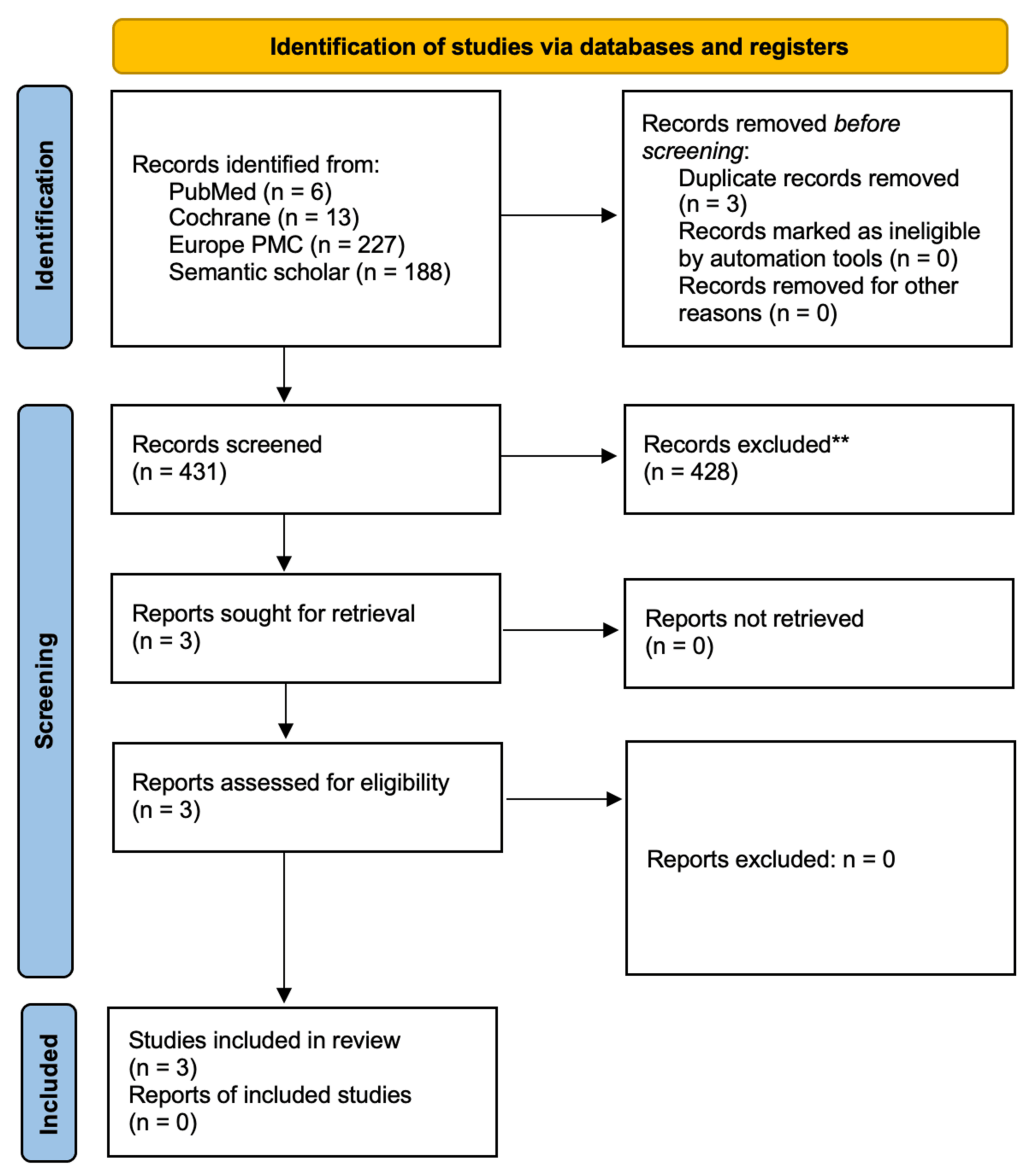
Preferred Reporting Items for Systematic reviews and Meta-Analyses (PRISMA) flow diagram

A total of 13 studies were identified through database searches (PubMed: 6, Cochrane: 13, Europe PMC: 227, Semantic Scholar: 188). After removing three duplicates, 431 studies were screened for eligibility. Following full-text screening, three studies met the inclusion criteria and were included in the final analysis. The PRISMA flow diagram (Figure 1) details this selection process.

Study Characteristics

The included studies consisted of three RCTs with a total of 289 participants. The interventions included non-selective beta-adrenergic antagonists (timolol), prostaglandin analogues (travoprost and latanoprost) and prostamides (bimatoprost). Table 2 summarises key characteristics of the included studies [14-16].

**Table 2:** Summary of key characteristics of the included studies. CCT: central corneal thickness, HT: hypertension, IOP: intraocular pressure, VA: visual acuity

Study	Design	Diagnosis	Population	Sample size of Black participants	Central corneal thickness measurement taken	Other data extracted	Washout period present	Diurnal variation of IOP consideration	Intervention(s)	Primary outcomes
Mansberger et al. [14]	Randomised controlled trial	Ocular hypertension	African American (+ White)	184	Yes	Age, CCT, Sex, Use of oral anti-hypertensives, history of diabetes and HT	No	No	Nonselective beta-adrenergic antagonists (126) + prostaglandin analogues (58)	IOP after 4-6 weeks
Noecker et al. [15]	Randomised controlled trial	Ocular hypertension and open angle glaucoma	Black	94	No	Adverse effects, biomicroscopy, ophthalmoscopy, VA, age, sex, eye colour, prior treatment	Yes (6 weeks)	Yes (10am +/- 1 hour)	Bimatoprost 0.03% + Travoprost 0.004% (prostamide vs prostaglandin analogue)	IOP after 3 months
Kitnarong et al. [16]	Randomised controlled trial	Ocular hypertension and primary open angle glaucoma	Black (+ White)	11	No	Eye colour (graded), age, sex, adverse effects (ocular and systemic)	Yes (2-4 weeks)	Yes (8am to 10am)	Latanoprost + timolol	IOP after 6 weeks (2 cycles cross-ver)

Risk of Bias Assessment

**Figure 2:**
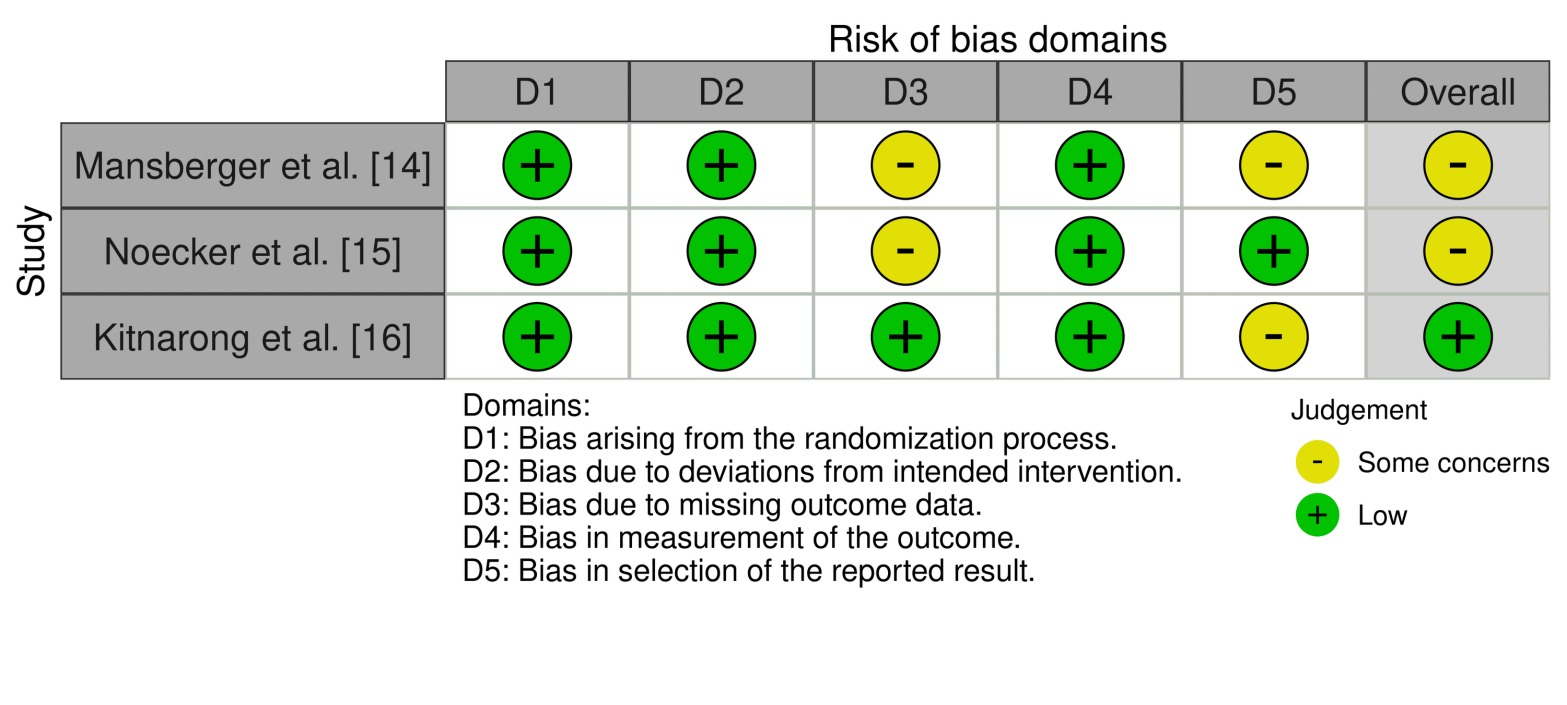
Graphical presentation of the risk bias assessment using the RoBVis visualisation tool.

The risk of bias assessment using the Cochrane RoB 2 tool indicated that one study had a low risk of bias, while two studies had some concerns due to missing data and selective reporting. Table 3 summarises the key aspects of the five domains in each study and Figure 2 provides a visual representation of the risk-of-bias assessment.

**Table 3:** Summary of the five domains as in the Cochrane RoB 2 for each study. CCT: central corneal thickness, RCT: randomised controlled trial, IOP: intraocular pressure

Bias Domain	Mansberger et al. (2007) [14]	Noecker et al. (2006) [15]	Kitnarong et al. (2004) [16]
Randomization Process	RCT, well-described allocation with no selective allocation.	RCT, randomised and investigator masked.	Double-masked, randomised crossover design.
Deviations from Intended Interventions	Investigator-masked, standardized protocol, controlled diurnal variation.	Investigator-masked, controlled diurnal variation.	Double-masked protocol, crossover study.
Missing Outcome Data	85% in treatment group completed follow-up, but 54% in observation group. No detailed handling of missing data.	No explicit mention of dropout rates or missing data handling.	Only two patients (three eyes) excluded for noncompliance or disease progression, no major protocol violations.
Measurement of Outcomes	Goldmann tonometry, blinded examiners, standardized CCT measurements.	Standardized IOP measurements, proper blinding.	Goldmann tonometry used, IOP measured consistently at 8 AM and 10 AM.
Selection of Reported Results	Stepwise multiple regression analysis could introduce selective reporting bias.	All pre-specified outcomes were reported, with no evidence of selective reporting.	Small sample size may have limited statistical power, but no strong evidence of selective reporting.
Overall Risk of Bias	Missing data, potential selective reporting, but overall well-conducted.	Limited transparency in missing data handling and reporting.	Strong methodology, minimal missing data, but small sample size.

Findings on IOP Reduction

All included studies measure IOP reduction as the primary outcome. Table 4 and Table 5 shows the interventions used in each study and their associated baseline IOP, final IOP, change in IOP and p-values. The exact term used in each study to describe the IOP-lowering agent such as 'non-selective beta-adrenergic antagonists', 'prostaglandin analogues', 'Bimatoprost', 'Travoprost', 'Timolol' and 'Latanoprost' has been used to summarise the findings.

**Table 4:** Summary table of the baseline IOP, final IOP and change in IOP for each intervention in Mansberger et al. and Noecker et al. IOP: intraocular pressure

	Intervention	Baseline IOP (mmHg)	Final IOP (mmHg)	Change in IOP (mmHg)	P-value
Mansberger et al. [14]	Non-selective beta-adrenergic antagonists	25.8 ± 3.0	19.6 ± 2.8	6.2	Not reported
	Prostaglandin analogues	25.3 ± 3.9	17.5 ± 3.8	7.8	
Noecker et al. [15]	Bimatoprost	25	17.1	6.8 - 7.8	p >/= 0.130
	Travoprost	24.5	17.7	6.2 - 6.9	

**Table 5:** Summary table of the baseline IOP, final IOP and change in IOP for each intervention in Kitnarong et al. IOP: intraocular pressure

	Time	Intervention	Baseline IOP (mmHg)	Final IOP (mmHg)	Change in IOP (mmHg)	P-value
Kitnarong et al. [16]	8 AM	Timolol	24.8±7.2	19.2±6.3	5.6±4.2	p = 0.013
		Latanoprost	23.4±6.8	16.1±3.7	7.3±5.9	
	10AM	Timolol	24.2±7.6	17.1±3.7	7.1±6.2	p = 0.19
		Latanoprost	25.8±6.1	15.8±3.1	10.2±7.0	

The comparison in IOP reduction between non-selective beta-adrenergic antagonists and prostaglandin analogues was not measured in Mansberger et al. [14], however they found that there was a slightly greater IOP response in the African American population compared to the White population. Noecker et al. [15] found that patients treated with bimatoprost had larger IOP reductions compared to patients treated with travoprost. Kitnarong et al. [16] found that latanoprost was more effective in lowering IOP compared to timolol in Black patients.

Adverse Effects and Safety Profile

Only Noecker et al. [15] and Kitnarong et al. [16] reported the adverse effects of their interventions. The various adverse effects specific for the interventions used have been reported in the table below. Table 5 summarises the adverse effects stated for each intervention in the three studies.

**Table 6:** Summary of the adverse effects recorded for each intervention in the three studies.

	Mansberger et al. [14]		Noecker et al. [15]		Kitnarong et al. [16]	
Intervention	Non-selective beta-adrenergic antagonists	Prostaglandin analogues	Bimatoprost	Travoprost	Timolol	Latanoprost
Adverse effects	Not reported	Not reported	Ocular redness (20.4%), itching (2.04%), grittiness (2.04%), collartettes (2.04%), bacterial conjunctivitis (2.04%), urticaria (2.04%)	Ocular redness (15.6%), itching (4.46%), foreign body (2.23%)	Stinging, conjunctival hyperaemia, blurred vision	Conjunctival hyperaemia, ocular burning

Discussion

Summary of Key Findings

In this study, we compared three existing randomised-controlled trials to evaluate the efficacy of IOP-lowering agents in Black patients. Mansberger et al. [14] and Kitnarong et al. [16] compared a non-selective beta-adrenergic antagonist with a prostaglandin analogue, while Noecker et al. [15] compared a prostamide with a prostaglandin analogue. Both Mansberger et al. [14] and Kitnarong et al. [16] found prostaglandin analogues to be more efficient in lowering IOP in Black patients compared to non-selective beta-adrenergic antagonists. Noecker et al. [15] found prostamides to be more efficient compared to prostaglandin analogues. Overall, prostaglandin analogues and prostamides have been found to be efficient in lowering IOP.

Comparison With Existing Literature

The NICE guidelines [17] have recommended prostaglandin analogues as first line ongoing treatment for patients with primary open angle glaucoma and ocular hypertension. Our study findings are in line with the national guidelines for the United Kingdom. Non-selective beta-adrenergic antagonists, non-generic prostaglandin analogue, sympathomimetic, carbonic anhydrase inhibitor, miotic or a combination of these treatments are recommended if treatment with prostaglandin analogue is unsuccessful or nor tolerated. Based on the findings of our study, the comparison between prostamide and prostaglandin analogue in Noecker et al. [15] found similar efficacies in both interventions in lowering IOP, with prostaglandin analogue being slightly more efficient. Taking this into account, there is potential for prostamides to be considered as a second-line treatment instead of beta-blockers in Black patients. Additionally in our study, beta-blockers have been proven to show limited efficacy in lowering IOP in Black patients compared to prostaglandin analogues.

Topical beta-blockers can be absorbed into the system causing systemic adverse effects such as dizziness, bradycardia, dyspnoea, complete atrioventricular block and bronchospasm. Given the high prevalence of undiagnosed cardiovascular and respiratory conditions in the African American population due to limited access to healthcare, utilising beta-blockers to treat glaucoma poses a potential risk in exacerbating these underlying conditions [18,19]. If beta-blockers were to be used instead of prostaglandin analogues, a 0.1% formulation would be a safer alternative due to reduced systemic absorption leading to lesser systemic adverse effects but with similar efficacy compared to higher concentrations, such as the widely used 0.5% formulation [20].

Clinical and Practical Implications

With increasing prevalence and severity of glaucoma in the Black community, optimising treatment strategies, medical, lasers, and surgical, is crucial to control disease progression and prevent vision loss. Our study confirms that the first-line ongoing treatment, a prostaglandin analogue, as recommended by the NICE guidelines is a suitable and efficient treatment in the Black community. Acknowledging the limited efficacy and unfavourable adverse effect profile of non-selective beta-adrenergic antagonist in Black patients, it would be more appropriate as a second-line treatment in Black patients where treatment with prostaglandin analogue is unsuccessful or not tolerated.

NICE guidelines have recently adopted findings from the LIGHT trial, resulting in a recommendation that selective laser trabeculoplasty (SLT) should be offered as an initial treatment for patients with ocular hypertension. Whilst the purpose of this systematic review is to investigate the most efficient IOP-lowering medical treatment, further studies should attempt to investigate and compare the efficacies between SLT and the IOP-lowering agents especially in the Black community. This is especially important in this community due to the lower adherence to daily eye drop regimens. SLT offers a quick solution which reduces the need for a daily treatment regime. However, the cost-effectiveness of SLT in this community needs to be studied [17,21].

Study Limitations

Despite the strengths of the study, there are several limitations to the study. The three studies had only investigated three classes of IOP-lowering agents, prostaglandin analogues, non-selective beta-adrenergic antagonists and prostamides. Therefore, there is reduced reliability in suggesting prostaglandin analogue to be the most efficient IOP-lowering agent for this population. Black patients are known to have thinner corneas which tends to underestimate IOP measurements [22]. Only Mansberger et al. [14] measured central corneal thickness. The lack of central corneal thickness in the Noecker et al. [15] and Kitnarong et al. [16] studies diminish the reliability of IOP measurements. Another limitation is the lack of investigation of confounding factors that may affect IOP levels, such as medication adherence and equipment used to measure IOP and central corneal thickness. Furthermore, the sample size in Kitnarong et al. [16] which only studied the efficacy of the interventions in 11 Black patients was another significant limitation in our study.

Future Directions

In future studies, it is crucial to involve a bigger sample size of both native and immigrant Black patients [23]. In addition to a larger sample size, a more rigorous technique in assessing the efficacy of various IOP lowering agents should be incorporated. This would be to utilise standardised equipment across all settings, measure central corneal thickness, include all classes of IOP-lowering agents, amount of iris pigmentation, assess medication adherence and report all adverse effects. Understanding the impact of all these confounding factors may aid in the development of an evidence-based Black patients’ specific guideline for treating primary open angle glaucoma and ocular hypertension.

## Conclusions

In conclusion, our study supports the current recommendation stated in NICE guidelines for prostaglandin analogues to be used as first-line treatment for primary open angle glaucoma and ocular hypertension in the Black community. However, more research is needed to optimise the second-line treatment or even assess if other classes of IOP-lowering agents may be more efficient than the current first-line treatment. By optimising therapy selection, clinicians can improve glaucoma management and prevent loss of vision in this vulnerable patient population.
